# Immunohistological analysis of CD1a^+^ langerhans cells and CD57^+^ natural killer cells in healthy and diseased human gingival tissue: A comparative study

**DOI:** 10.4103/0972-124X.60228

**Published:** 2009

**Authors:** Sahaya Stelin, Hemalatha Ramakrishan, Avaneendra Talwar, K. V. Arun, T. S. S. Kumar

**Affiliations:** *Department of Periodontics, Ragas Dental College and Hospital, Uthandi, Chennai, India*

**Keywords:** CD1a^+^ Langerhans cells, CD57^+^ natural killer cells, human gingival tissue

## Abstract

**Background:**

Cell interaction between dendritic cells (DC) and natural killer (NK) cells in the periodontal milieu is not yet fully known, although these cells are individually known to contribute to the pathogenesis of periodontal disease.

**Materials and Methods:**

Fifty subjects (25 males and 25 females) were included in the study. The subjects were divided into three groups: Group A comprised 16 subjects with clinically healthy gingiva; group B 17 subjects with gingivitis; and group C 17 subjects with gingivitis; and group C 17 subjects with moderate periodontitis (PPD ≥ 5 mm and CAL ≥ 3 mm in at least six sites). Gingival samples were collected and immunohistochemical study was done using CD57 and CD1a antibody. Statistical analysis was done using analysis of variance (ANOVA), followed by Tukey-Kramer multiple comparison for CD1a and Tukey's highly significant difference (HSD) test for CD57.

**Results and Conclusion:**

The study showed an inverse relationship between the CD1a^+^ (langerhans) cells and CD57^+^ (natural killer) cells. There was a significant increase in CD57^+^ cells and reduction in CD1a levels as periodontal disease progressed. The significant reduction in CD1a levels in periodontal disease when compared to health could possibly be a result of NK cells down regulating it. Reduction in CD1a levels may result in a low inflammatory response subsequently resulting in tissue destruction.

## INTRODUCTION

Periodontal diseases are thought to result from a dysregulation in the immune response of the host to putative periodontal pathogens.[1] Bacteria and bacterial products may enhance or inhibit the effector and regulatory activity of human lymphocytes. Antigen presenting cells (APC) are required to present the microbial antigens for the recognition and subsequent activation of T lymphocytes.[2]

Langerhans cells (LCs) are APC which belong to the dendritic cell subset that are chiefly distributed in the suprabasal layer in the gingival epithelium. On exposure to foreign antigen (Ag), LCs migrate to the connective tissue and present these Ags to the lymphocytes.[3] Seguier[4] reported that LCs decrease in the epithelium with gingivitis and periodontitis.

Natural killer (NK) cells constitute a distinct lineage of cytotoxic lymphocytes which constitute a major component of the innate and cell mediated immune response. These cells develop in the bone marrow and lack rearranged T cell receptors (TCR). They have the ability to kill target cells without prior sensitization by direct contact with the cell and produce proinflammatory cytokines, especially interferon gamma. These cells are essential not only for defense against pathogens but also for the initiation of adaptive immune response and in regulating autoimmune mechanisms.[5]

The role of NK cells in oral diseases has not been fully established. Tsoumis[6] demonstrated a significant positive correlation between peripheral blood NK cytotoxicity and degree of inflammatory periodontal disease. Fujita[7] reported an increased infiltration of the gingival connective tissue by NK cells in severe forms of periodontal disease and suggested that these cells may play a role in the destruction of tissues in periodontal disease.

Various studies have demonstrated a relationship between NK cells and dendritic cells (DCs).[8–10] The NK cell possess surface receptors (activating and inhibitory receptors) required for the recognition of self (HLA class I expressing cells) from infected cells when in contact with the target cells. These interactions are thought to be modulated by the CD1a molecules expressed on the surface of LCs.[10] This interaction can mediate cytotoxic T cell differentiation and NK cell activation/priming.[11]

NK cells and DCs may demonstrate an inverse relationship to each other with regard to their activity. Abruzzo and Rowley[12] suggested that NK cells may down-regulate antibody response by eliminating APCs while displaying antigen. Carbone[13] reported that the expression of CD1 by DCs can inhibit NK cell mediated lysis. Buentke[9] showed that NK cells and CD1a^+^ DCs were in close contact in atopic dermatitis syndrome.

The interaction between these cells in periodontal disease is yet to be fully elucidated.

The present study was undertaken to compare the levels of CD57^+^ NK cells with CD1a^+^ LCs in healthy and diseased gingival tissues.

## MATERIALS AND METHODS

### Subjects

A total of 50 subjects (25 males and 25 females) selected from the patient pool attending the outpatient clinic, Department of Periodontics, Ragas Dental College and Hospital, were enrolled in the study between January and June 2006. The Institutional Review Board of Ragas Dental College and Hospital approved the study protocol and informed consent was obtained from each subject. Subjects exhibiting good general health with no history of smoking or periodontal treatment or antimicrobial therapy for the past six months were chosen for the study.

The subjects were divided into three groups: Group A comprised 16 subjects (nine males and seven females; age: 22 to 34 years; mean, 30.7 years) with clinically healthy gingiva (probing pocket depth (PPD) <3 mm; no clinical attachment loss (CAL); no bleeding on probing); group B comprised 17 subjects (eight males and nine females; age: 31 to 41 years; mean, 35.5 years) with gingivitis (PPD ≤ 3 mm; no CAL; presence of bleeding on probing); group C comprised 17 subjects (eight males and nine females; age: 36 to 45 years; mean, 39.5 years) with moderate periodontitis (PPD ≥ 5 mm and CAL ≥ 3 mm in at least six sites).

### Tissue collection

Gingival samples were collected from subjects in group A while they underwent extraction for orthodontic reasons or during third molar removal; from subjects in group B during extraction of teeth for reasons other than periodontal pathology; from subjects in group C while they underwent extraction of teeth which had hopeless prognosis due to periodontal pathology. The tissues obtained from the subjects were fixed with 10% neutral buffered formalin and then embedded in paraffin within 48 hours to avoid antigen degradation. Serial sections that were 4 μm thick were made from the specimens and mounted on 3-aminopropyltriethoxysilane coated slides.

### Immunohistochemistry

The tissues were deparaffinized by two changes in xylene (10 minutes each), put in descending grades of alcohol and then rehydrated with water. They were then transferred to citrate buffer and antigen retrieval was done using microwave at power 4 for 30 minutes. Then the slides were dipped in two changes of phosphate buffered saline (PBS) for 5 minutes and then wiped carefully with gauze to remove excess PBS. The slides were treated with 3% hydrogen peroxide for 10 minutes, put in two changes of PBS and then treated with power block for 20 minutes.

The primary antibodies mouse monoclonal anti-human CD57 antibody (BioGenex) and CD1a antibody (BioGenex) were added to different tissue sections of the same tissue. The petridish containing the slides was kept at room temperature for one hour. After one hour, the sections were taken out and washed in two changes of cold PBS (10 minutes each), a drop of super enhancer added and the slides incubated for 30 minutes. The slides were then washed in two changes of cold PBS (10 minutes each). A drop of polyhorseradish peroxidase (HRP) was added onto the sections and the slides incubated for 30 minutes. The sections were then washed in 2 changes of cold PBS for 10 minutes each. The slides were wiped carefully to remove excess PBS and a drop of freshly prepared DAB (3′-Diamino benzidine Tetra Hydrochloride—a substrate Chromogen) was added onto the sections. Slides were then washed in running distilled water to remove excess DAB and counter stained with Harris's Hematoxylin. They were then washed with acid alcohol and xylene. The tissue sections were mounted with disterene dibutyl phthalate (DPX) for microscopic examination at magnification ×10 and ×40.

### Evaluation

CD 57 and CD1a expression were evaluated by counting the number of cells that were stained. The percentage of positive cells was the ratio of the number positively stained cells divided by the total number of cells counted. A total of 1,000 cells were counted in each slide. Thus,

Labelling index (LI) = (number of positive cells/1,000) × 100.

### Statistical analysis

Data entry and descriptive analysis was performed using the SPSS statistical software program. Analysis of variance (ANOVA), followed by Tukey-Kramer multiple comparison test, was used to compare the groups with regard to CD1a antibody. ANOVA followed by Tukey's highly significant difference (HSD) test was used to compare the groups with regard to CD57 antibody.

ANOVA followed by Tukey's highly significant difference (HSD) test was used to compare the groups with regard to CD57 antibody.

## RESULTS

All tissues examined showed the ˋpresence of oral gingival epithelium and subjacent connective tissue. All the positive cells stained brown. The results are summarized in Table 1.

**Table 1:** Mean labeling index for CD1a^+^ langerhans cells and CD57^+^ natural killer cells in healthy and diseased gingival tissue

Group	CD 1a^+^ (mean ± SD) %	CD57^+^ (mean ± SD) %
Healthy (group A)	75.71 ± 3.09	3.89±1.64
Gingivitis (group B)	42.53 ± 3.88	18.47 ± 2.45
Periodontitis (group C)	29.07 ± 3.68	33.13 ± 3.11
*P* value	<0.001*	<0.001*

* Statically significant

### Mean labeling index of CD1a

**Figure 1:**
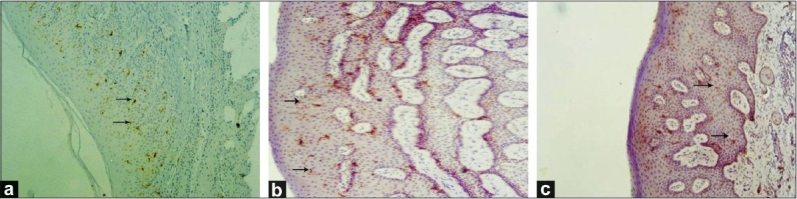
Expression of CD1a^+^ cells in (a) healthy gingiva, (original magnification, ×10); (b) gingivitis, (original magnification, ×40); (c) periodontitis (original maginification, ×40) group. Intense brown cytoplasmic staining seen in the epithelium (denoted by black arrows), with no staining in the connective tissue. CD1a^+^ cells indicated by arrow

An intense cytoplasmic staining was observed in supra basal layers of the gingival epithelium in the healthy group (group A); whereas in the gingivitis (group B) and periodontitis (group C) groups, the staining was milder. In the healthy group, the suprabasal cells showed increased reactivity when compared to basal portions. No staining was observed in the connective tissue [Figure 1a–c].

The mean labelling index in individual groups was: Group A, 75.70 ± 3.09; group B, 42.53 ± 3.09; and group C, 29.07 ± 3.08. There was a statistically significant difference between all the groups (*P* value < 0.001).

### Mean labeling index of CD57

An intense brown cytoplasmic staining was observed subjacent to the epithelium. The number of positively stained cells was fewer in the healthy gingival group (group A) compared to the gingivitis (group B) and periodontitis (group C) groups.

**Figure 2:**
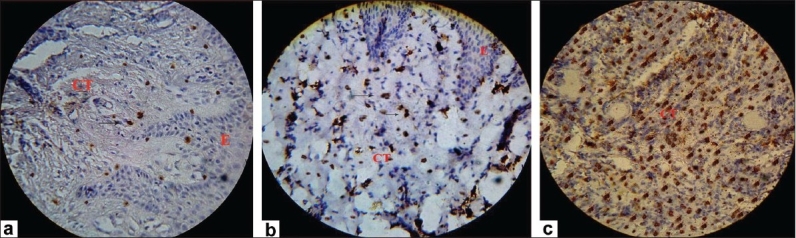
Expression of CD57^+^ cells in (a) healthy gingiva; (b) gingivitis; (c) periodontitis group. CD57^+^ cells indicated by arrow (original magnification, ×40)

The mean LI in individual groups was: Group A, 3.87 ± 1.64; group B, 18.47±2.45; and group C, 33.13±3.11. There was a statistically significant difference between all the groups (*P* value <0.001) [Figure 2a–c].

## DISCUSSION

DCs are capable of engaging and internalizing a wide variety of pathogens, and upon endocytosis, they can process antigens and prime T lymphocytes and initiate adaptive immune response.[14] LCs belong to the skin resident member of the dendritic family of APCs. They initiate both innate and adaptive immune responses to relevant antigens, thereby acting as immunological sentinels. LCs express CD1a molecules at exceptionally high levels with virtually no detectable CD1b and only modest CD1c expression, whereas other DC subsets predominantly display CD1b molecules with varying degrees of CD1a and CD1c expression.[15] LC-like DCs are more efficient at presenting nonpeptide antigens to *t*-cells via CD1a than monocyte derived dendritic cells.[16]

CD1a which was used as a marker of LCs in the present study belongs to group1 CD isoform which are capable of presenting various forms of self and microbial lipid antigens to T lymphocytes. Following lipid antigen capture, LCs migrates to regional lymphnodes. Here, the CD1a molecules are transported from the cell surface to early recycling vesicles and Birbeck granules. In the Birbeck granules, CD1a is coupled with internalized lipid moieties and presented to *T*-cells. During this process of antigen presentation, LCs loses the CD1 molecule on their surface.[15] The induction of CD1a expression can be mediated by cytokines (IL-1, GM-CSF, TNF-α, and IL-6) secreted by activated keratinocytes and monocytes.[17] The location of LCs in the epidermis allows the innate immune system to respond rapidly to microbial invaders in the epithelium and result in improved clearance of the organism.[16]

The present study suggests that there was a decrease in CD1a expression in periodontal disease. These findings are in conformity with previously published reports.[4,18] LCs are believed to migrate into the connective tissue following antigenic stimulation such as that which occurs in periodontal disease.[19] The fate of these cells after they reach the connective tissue is not yet fully determined. While some authors suggest that they may migrate to the regional lymphnodes for T cell activation,[20] others have reported formation of ectopic lymphoid organ within the gingiva.[21] Either way, their migration into the connective tissue provides an explanation for the decrease in LC expression in gingival epithelium. In contradiction, Gemmel[22] demonstrated the presence of numerous CD1a^+^ LCs in the gingival epithelium with no differences between the healthy/gingivitis and periodontitis groups. Cutler and Jotwani[18] proposed a role of DCs in periodontal disease by activating a destructive T cell response mediated by both regulatory and effector T cells, in response to pathogenic bacteria.

NK cells are lymphocytes of the innate immune system that are involved in the early defense against foreign cells and microbial infection. Upon stimulation, NK cells secrete large amounts of cytokines and chemokines necessary to control bacterial and viral infections. NK cells are also cytotoxic, inducing apoptosis of cells recognized as targets.[10]

Expression of CD16, CD56, and CD57 on the NK cell determine their activation status. CD57 which was used as a marker of NK cells in the present study, also known as beta-1,3-glucuronyltransferase 1 (B3GAT1), human natural killer (HNK-1), NK-1, and Leu-7 is a 100-115kD oligosaccharide antigenic determinant expressed on extracellular proteins of certain cell types. CD57 is expressed on a subset of 15-20% of peripheral blood mononuclear cells, including 60% active NK cells and CD8^+^ T cells and is also expressed on neural cells and striated muscle. CD57 is not expressed on red cells, granulocytes, monocytes, or platelets. While the function of CD57 is unknown, binding to L-selectin, P-selectin, and a fragment of laminin suggests that CD57 may be involved in cell-matrix interactions. CD57 is increased in some disease states associated with CD4/CD8 imbalances.[23,24] A CD57^+^ only cell denote a weakly active natural killer cell.

The results of the present study indicate increased levels of CD57^+^ NK cells in gingival tissues as disease progresses. This finding is in conformity with previously published results.[7,25,26] Tsoumis,[6] demonstrated a significant positive correlation between peripheral blood NK cytotoxicity and degree of inflammatory periodontal disease. The precise role played by these cells in modulating periodontal disease is not fully understood. The greater presence of these cells in periodontal tissues could be due to their greater availability and access to the site of inflammation.

In the present study, we showed that there is an inverse relationship between CD1a^+^ LCs and CD57^+^ NK cells as periodontal disease progresses. This could be due to the elimination of APC by NK cell,[12] which in turn results in suppression of the inflammatory response, resulting from failure of Ag presentation. Inflammatory responses are normally meant to help in clearing pathogens. However, low level responses that fail to clear a significant portion of the bacteria could result in a sustained immune response and promote tissue injury. Moreover, CD1 positive cells are known to suppress NK cells cytotoxic activity. The decrease of CD1 positive cells removes this inhibitory signal.[13]

The results of the present study suggest that these cells may adversely affect the function of each other, thereby modulating the pathogenesis of periodontal disease.
